# Drivers of fishermens’livelihood resilience and transitioning to ecological farming in Yangtze River,China:From PLS-SEM and fs/QCA

**DOI:** 10.1371/journal.pone.0338704

**Published:** 2025-12-10

**Authors:** Xueming Wang, Jianming Zheng, Tinggui Chen

**Affiliations:** 1 School of Economics and Management, Shanghai Ocean University, Shanghai, China; 2 Yangtze River Aquatic Ecosystem Protection Strategic Research Center of Ministry of Agriculture and Rural Affairs of the Peoples’s Republic of China, Shanghai, China; Flinders University, AUSTRALIA

## Abstract

This study investigates the effects of risk perception, buffering ability, self-organization ability and learning ability on the fishermens’ecological farming willingness in Yangtze River, China. Using the Yangtze River basin fishermen as a case study, this study simultaneously used the partial least squares structural equation model (PLS-SEM) and fuzzy-set qualitative comparative analysis (fs/QCA) to explore the linear and nonlinear dynamic impacts among the variables. PLS-SEM analysis revealed that livelihood resilience positively affect ecological farming willingness of retired fishermen; risk perception and livelihood resilience can positively influence ecological farming willingness through buffering, self-organization and learning abilities. The fs/QCA revealed that simple antecedent variables do not constitute a necessary condition for promoting fishermens’ strong ecological farming willingness, which depends on the conditions combined with another element. The analysis also elucidates the optimized combination of support policies for fishermen in different regions. In the eastern region, a compensation configuration that emphasizes “livelihood capital” through natural and material resources is identified as the most effective approach. This strategy aims to enhance the stock of livelihood capital for fishermen, thereby ensuring stability and sustainability in their livelihoods. Conversely, the central region is most effectively served by “policy-driven” assistance measures. These measures emphasize enhancing policy implementation to promote the transformation and restoration of livelihoods. Simultaneously, in the western region, an optimal comprehensive support model, characterized as “integrated capital -policy-community-skills,” comes to the fore. This approach empowers communities and offers substantial support for the sustainable development of fishermen’s livelihoods.

## 1. Introduction

The implementation of a ten-year fishing ban in the Yangtze River represents a crucial decision made by the Central Committee of the Communist Party of China and the State Council [1]. This decision is based on long-term strategic interests and the pursuit of a more prosperous future for future generations [2]. It indicates a significant progress in the protection and management of major river systems [3,4]. The “No.1 Central Document” issued in 2021 emphasized the importance of ensuring the effective implementation of this long-term fishing ban by offering necessary support to retired fishermen [5]. Since the full implementation of the “ten-year fishing ban policy”, approximately 164,500 river fishermen in need of re-employment have received necessary assistance, and around 221,800 eligible retired fishermen have been ensured insurance coverage [6]. As the indigenous inhabitants of the Yangtze River basin, fishermen experience substantial transformations in their livelihoods and living environment upon relocation to land. Their adaptation to the new social identity is still in the process of development. The fishing moratorium in the Yangtze River has led to the loss of the fishermen’s primary source of livelihood. During the transitional phase from the implementation of the fishing ban to the shift towards alternative occupations, fishermen are especially susceptible to disruptions in their livelihood system. Although policies aim to restore species diversity and community structure, and ensure sustainable livelihood of fishermen, the effectiveness of these policies depends on multiple factors such as the fairness of policy formulation, as well as the perceptions, attitudes, and compliance of fishermen. Consequently, there is an immediate necessity to identify a livelihood transformation approach that not only enables straightforward operations to assist fishermen in rapidly reinstating their livelihoods but also contributes to watershed ecological conservation and fisheries development.

The advantages of adopting ecological farming livelihoods are manifested in their low capital investment requirements, high potential for sustainability, and suitability for fishermen with advanced age and lower educational levels [1,7]. The fishermen’s tendency to engage in ecological farming essentially represents a behavioral change in their livelihood strategies. During this transitional period, the fishermen’s ability to confront challenges directly influences the strength of their inclination to embrace innovative practices. The concept of livelihood resilience emphasizes the capacity of individuals to formulate adaptive strategies in the face of shocks within a vulnerable context [8]. As such, the introduction of the concept of livelihood resilience aids in understanding the fundamental motivations underlying fishermen’s adoption intentions. Ifejika et al. have put forward an indicator system for assessing household livelihood resilience [9]. Moreover, numerous scholars have carried out comprehensive investigations into the various factors that impact household livelihood resilience. In the realm of livelihood strategy selection, enhancing livelihood resilience promotes households’ tendency towards non-agricultural livelihoods [10]. Regarding economic decision-making, increased livelihood resilience supports rural migrants’ urbanization intentions, stimulates venture capital undertakings [11], and encourages responses to land withdrawal policies [12]. Although research on livelihood resilience has advanced at a rapid pace, the majority of studies predominantly focus on measuring and analyzing it within specific external perturbations. There remains a gap in exploring how livelihood resilience affects the behaviors of fishing families.

The livelihood situation of fishermen is not only dependent on the degree of external disturbance pressures but also on their individual resilience and ability to recover from the resulting adversities [13].As an essential part of the sustainable livelihood theory [14], it focuses on the dynamic evolutionary process and mechanisms of livelihoods, highlighting the strategic utilization of limited resources by farmers to adjust to and adapt to external disturbances [15]. The theoretical framework of livelihood resilience can be categorized into three primary groups: a singular framework centered on the relationship between livelihood capital formation and community disaster recovery [16], a multidimensional analysis framework emphasizing resilience and adaptability [17], and a comprehensive analysis framework encompassing buffering, self-organization, and learning abilities [18]. In this research, livelihood capital is employed as the primary indicator of buffering ability, and the framework is constructed from the perspectives of the actors themselves and their interactions with social organizations. Consequently, a livelihood resilience assessment system emphasizing buffering ability, self-organization ability, and learning ability is established [19]. Integrating livelihood resilience into the analysis of fishermen’s propensity to adopt alternative livelihoods during fishing moratoriums provides a more comprehensive explanation of its formation rationale. Theoretically, fishermen with strong livelihood resilience are more capable of overcoming the challenges faced in adopting new livelihoods.

The implementation of ecological farming has the potential to generate increased profits. However, it also introduces specific technical impediments and market uncertainties [20]. Specifically, ecological farming is distinguished by a high degree of integration and intrinsic market risks. This requires practitioners to closely monitor market trends to guarantee access to high-quality information for production. Moreover, ecological farming demands the comprehensive implementation of fertilization and pest management techniques, thus presenting a certain level of technical threshold [21]. The different extents of risk perception among practitioners affect their propensity to adopt integrated farming practices, which is based on their assessment of associated risks and their stances towards potential high returns and possible losses [22]. Consequently, does the resilience exert an impact on practitioners’ willingness to adopt these practices? Furthermore, is there a significant difference in the influence of livelihood resilience on adoption willingness across different levels of risk perception?

The Yangtze River Basin, located in China, is one of the world’s largest freshwater basins [3]. Only by making every effort to boost the vitality of fishing villages and increase fishermen’s incomes can a stable and solid social foundation for fishing bans be established.The marginal contributions of this study are presented as follows: Theoretical perspective: This study uncovers the intricate causal mechanisms between livelihood resilience and the inclination to adopt ecological farming. Conventional research has predominantly concentrated on linear associations and net impacts. Nevertheless, this study synthesizes the dual perspectives of “ability” and “perception” to establish a more elaborate explanatory framework. By incorporating risk perception as a moderating variable, it empirically substantiates its function in either “strengthening” or “impeding” the aforementioned causal pathways, thus deepening our comprehension of individual decision-making processes under risk circumstances.

Methodological perspective: This research attains a theoretical integration and reciprocal validation of symmetric and asymmetric analyses. Innovatively amalgamating PLS-SEM with fs/QCA for mixed-method research enables methodological cross-validation and complementarity [23]. PLS-SEM effectively examines both the net effects among variables and the moderating effects, guaranteeing the statistical robustness of the model. In contrast, fs/QCA surpasses symmetrical logic by disclosing multiple equivalent antecedent configurations that prompt a high level of adoption willingness. Practical perspective: The findings offer targeted evidence for formulating precise and differentiated environmental policies. The conclusions go beyond general policy guidelines focused on “enhancing livelihood resilience.” Instead, they advocate for the design of intervention measures that integrate diverse tools rather than relying on a single approach to conform to the capital endowment characteristics of different fishing communities.

## 2. Theoretical analysis and research hypotheses

The ecological farming model has emerged as an innovative approach to agricultural production, which the Ministry of Agriculture and Rural Affairs has characterized as “a revolution in modern agriculture [22].” This model realizes the dual utilization of water resources, mitigates agricultural pollution, and elevates ecological benefits in the agricultural sector. It signifies a substantial improvement and transformation of conventional farming practices [24]. The residents of Qianjiang City, situated in Hubei Province, have adopted an ecological cycle model featuring the integration of “rice and fish.” In this model, rice plants provide shelter and microbial nourishment for fish, and simultaneously, the waste produced by fish serves as a natural organic fertilizer [25]. This methodological approach not only reduces the dependence on chemical fertilizers and pesticides but also enhances the quality of both rice and fish [20]. In Louisiana, USA, fishermen have achieved a concurrent increase in both yield and net profit through the mechanized and large-scale cultivation of crayfish and rice [26]. In Thailand, fishermen have gained a competitive advantage via organic certification, leading to a 42% reduction in carbon emissions compared to traditional systems [27].

There are three principal considerations to be addressed. Initially, ecological farming demands a series of initial investments, including the procurement of seedlings and fertilizers for cultivation. The long non-productive periods characteristic of certain crops exacerbate the financial difficulties faced by fishermen, particularly those with limited capital for their livelihoods [2,10]. Secondly, the ecological farming model requires a high level of technical proficiency. Consequently, fishermen are obliged to acquire and improve new capabilities. Only through specialized skill development and meticulous crop management can they achieve stable yields characterized by both high quantity and quality [11,19]. Thirdly, to maximize production returns in an uncertain market environment, fishermen must enhance their ability to collect and analyze information, thus attaining a deeper understanding of market dynamics [5].

### 2.1. The impact of livelihood resilience on retired fishermen’s willingness to adopt ecological farming

The concept of livelihood resilience refers to the ability of individuals and family units to withstand disruptions, maintain basic systems and practices through organizational endeavors and the acquisition of knowledge [28]. This research defines livelihood capital as a fundamental indicator of resilience and constructs a framework that integrates individual actions and their interactions with social organizations [15].

Initially, buffering ability is demonstrated through livelihood capital and dynamic processes [29]. This refers to the capacity of fishermen to maintain stable livelihoods in the face of sudden changes, based on their inherent resource endowments. It serves as a driving factor within the framework of livelihood resilience [8]. Buffering ability provides fishermen with a sense of livelihood security and helps mitigate the impacts of external shocks on their livelihoods, thus offering substantial support for the adaptation of their livelihood strategies [30]. Fishermen rely on these resource endowments to counter external risks, which are represented by five categories of capital: natural, material, social, financial, and human capital [21].

The self-organization ability is defined as the ability of fishermen to integrate into social structures, comply with institutional models, and understand the policy environment [31]. When faced with external disturbances, the self-organization ability enables fishermen to utilize social resources and policy support to enhance the stability of their livelihoods, thereby providing social support for the changes in their livelihood strategies [32]. Specifically, it examines how government subsidies, fishermen’s policy awareness, and the levels of trust among neighbors potentially influence fishermen’s propensity to adopt ecological farming practices. Participation in ecological farming requires corresponding financial incentives [33]. Therefore, the availability of subsidies significantly impacts fishermen’s inclination to participate. The subjective intentions of fishermen, who act as decision-makers, are intricately intertwined with their understanding of relevant policies [34]. Moreover, existing research demonstrates that rural villagers maintain interactions based on personal relationships that nurture trust and emotional connections [35,36]. This dynamic engenders a sense of identity and belonging among community members, which has the potential to stimulate prosocial behavior.

The capacity for learning endows individuals with the ability to rapidly reconfigure their livelihood strategies in the face of external perturbations, providing value – based and personal emotional support for the dynamic livelihood adjustments of fishermen [37,38]. Two conditions, namely technical training and information exchange, have been selected to assess the learning abilities of fishermen. Active engagement in training facilitates the development of a more comprehensive comprehension, reducing the uncertainties related to the application of novel skills. Consequently, it mitigates the adverse selection phenomena in ecological farming [15]. Moreover, information asymmetry can exacerbate the costs of information acquisition, affecting both the accuracy of government agricultural policies and fishermen’s propensity to actively participate in their livelihoods [39].

Buffering ability offers a margin for risk tolerance, thus mitigating concerns related to adoption [20]. The synergy with self-organization ability is demonstrated as follows: when fishermen have a certain degree of resource buffer, they are more inclined to participate in collective action, which, in turn, reduces individual adoption costs via resource sharing [17]. The synergy with learning ability is reflected in the fact that fishermen with abundant resources are better able to shoulder the costs of learning [5]. Furthermore, the results of this learning can enhance resource utilization efficiency, establishing a positive feedback loop [29]. The “collective resources” inherent in self-organizing ability can offset the limitations in individual buffering capacity [21], consequently enhancing the overall risk resilience and augmenting the inclination to adopt novel practices [40]. Furthermore, self-organizing platforms (e.g., field teaching) promote experience sharing, expedite the localization of technological knowledge adaptation [15], and mitigate hesitance arising from information asymmetry [39]. Lastly, the learning ability empowers fishermen to adjust resource allocation flexibly, thereby indirectly strengthening their resilience and facilitating sustained adoption [7,24]. Furthermore, collective learning, such as participatory technology development, can give rise to localized solutions that spread via social networks [9], generating a impetus for group adoption.

In general, the enhancement of self-organization and learning abilities is based on improving buffering ability, optimizing capital utilization, and realizing the effective transformation of livelihood capital [2]. Livelihood capital is essential for fishermen seeking diversified livelihood strategies, coping with external changes and uncertainties, and promoting livelihood transformation [15]. Under the existing buffering ability, increasing the emphasis on education and government policy support is vital to broadening the path of other abilities and ecological farming strategies. In light of the aforementioned analysis, this paper posits the subsequent research hypothesis:

H1: The three dimensions of livelihood resilience, namely buffering ability, self – organization ability, and learning ability, have a positive impact on fishermen’s propensity to adopt ecological farming. And the three abilities interact dynamically.

### 2.2. Analysis of the moderating effect of risk perception on the influence of livelihood resilience on adoption willingness

In the context of the absolute ban on fishing activities in the Yangtze River Basin, the suspension of fishing operations is bound to disrupt the traditional livelihoods of fishermen, thus having a significant impact on their economic well-being [3]. In the subsequent phase of rehabilitation and adaptation, fishermen are obligated to restructure their livelihood-related capital assets, adjust their subsistence strategies, and foster new sustainable living cycles [1,5]. This transition is unavoidably accompanied by specific risks related to livelihoods. Risk perception refers to an individual’s subjective ability to assess and understand risk-involved scenarios [40]. It reflects a decision-maker’s consistent propensities regarding choices made under different levels of risk or their preparedness to undertake such risks [41]. Given the significant disparities in individual risk perceptions, the influence of risk occurrences on decision-making processes also exhibits inter-individual variations [36]. Fishermen possessing superior cognitive abilities are more likely to effectively evaluate perceived risks and determine whether the adoption of ecological farming models is suitable for realizing a successful transformation of their livelihoods [29].

Although the implementation of ecological farming holds the potential to yield more substantial expected benefits, it is accompanied by intrinsic selection risks [42]. Specifically, in the initial stages of ecological farming, challenges may arise, including slow sales of ecological products, dependence on specific channels, and difficulties related to certification and trust [12,20]. Furthermore, fishermen are required to take into account climate sensitivity, the risk of ecological imbalance, as well as uncertainties related to subsidies and insufficient funding [27,37]. In the process of making decisions about adoption, fishermen assess the degree of risk involved [24]. Individual disparities in risk perception can either exert a facilitating or a constraining influence on the relationship between livelihood resilience and the willingness to adopt new practices [30]. Fishermen characterized by a strong ability for risk recognition are prone to exhibiting higher levels of psychological resilience [43]. They assume a more proactive approach to risks and are more capable of leveraging their livelihood capital, self-organization, and learning capabilities to address environmental changes [30]. This enhances the positive influence of livelihood resilience on their inclination to participate in ecological farming. Based on this comprehension, this paper puts forward the following research hypotheses:

In the event that fishermen become cognizant of the risks linked to environmental degradation (e.g., the deterioration of water quality and the decline of fish populations) or market fluctuations, they may take proactive measures to augment their savings or procure emergency assets [11]. When fishermen exhibit a certain degree of economic resilience, they are more prone to shoulder the initial expenses related to ecological farming (e.g.,procuring environmentally-friendly feed and establishing recirculating water systems) [13]. When fishermen acknowledge that individual endeavors may prove inadequate in addressing environmental or market risks, they tend to exhibit a greater propensity to participate in fishing cooperatives for the purpose of resource sharing [5]. Cooperatives have the potential to mitigate the costs associated with technology adoption for individual fishermen [7]. For instance, this can be achieved through the collective procurement of ecological feed and the unified certification of organic aquatic products [29]. When fishermen acknowledge the unsustainability of traditional fishing methods, they will exhibit greater initiative in learning techniques and participating in training programs provided by the government or non - governmental organizations (NGOs) [18]. Fishermen who acquire these techniques are more inclined to recognize the viability of ecological farming practices, such as the “aquaponics” model, which can mitigate pollution and improve profitability [19]. When all three abilities are fortified, the resilience of livelihoods is augmented, thereby elevating the fishermen’s inclination to participate in ecological farming. Nevertheless, in the event that any one of these abilities is deficient, such as inadequate financial resources, unorganized institutional frameworks, or deficiencies in technical expertise [21], it may give rise to a reduced willingness for ecological farming and constrain their recuperative potential.

H2: The correlation between livelihood resilience and the propensity to adopt ecological farming practices is affected by fishermen’s risk perception. In situations characterized by heightened risk awareness, the positive influence of livelihood resilience on the willingness to embrace these practices is strengthened.

**Fig 1:**
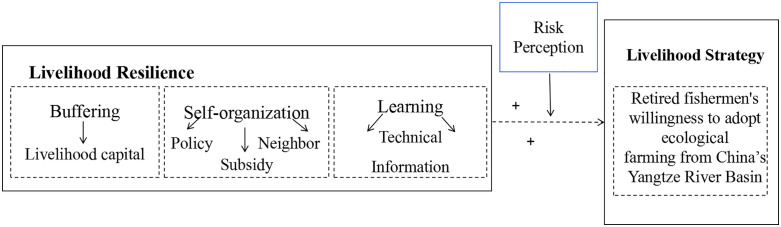
Research framework.

Beyond the depicted linear relationships, the concepts portrayed in Fig 1 may also include nonlinear connections. Consequently, our research adheres to two overarching principles:

P1: The willingness of fishermen to adopt ecological farming is influenced by various factors, not only a single factor.

P2: Thus, the driving forces of strong willingness do not fully contradict those that drive weak willingness.

In conclusion, risk perception, buffering ability, self-organization ability and learning ability are essential to the willingness of fishermen to adopt ecological farming. Based on the above assumptions, we use the PLS-SEM and fs/QCA to explore the mechanism affecting the resilience of fishermens’ livelihoods and reveal the configuration path promoting fishermens’ ecological farming willingness through the configuration effects of four antecedents: risk perception, buffering ability, self-organization ability and learning ability. The interactions of the model are illustrated in Fig 1.

## 3. Materials and methods

### 3.1. Study area and data sources

The dataset is sourced from the 2021 “Tracking Survey of Fishermen Affected by the Yangtze River Fishing Ban”. Considering the interrelationship between the living standards of fishermen and the economic development indicators of their respective regions, this study was designed to guarantee the authenticity of the research results and the uniformity of the sample data. Therefore, provinces with significantly high or low economic development indicators were excluded from the selection of survey regions. Based on the geographical divisions of the surveyed areas, the Yangtze River was divided into three main zones: the upstream zone consists of Sichuan, Chongqing, Yunnan, and Guizhou; the midstream zone includes Hunan, Henan, and Hubei; and the downstream zone encompasses Anhui and Jiangxi.

This study adopted a stratified random sampling approach to carry out household surveys among fishermen in the main (and tributary) conservation areas of the Yangtze River, non-conservation zones along the main (and tributary) rivers, and inner lake protected regions. In total, 409 fishing households were surveyed across multiple districts, towns, and villages. A total of 409 questionnaires were distributed. After eliminating non-responses and abnormal data, 397 valid questionnaires were retrieved, yielding an effective response rate of 97.07%. The survey covered the individual characteristics of fishing households, family endowments, as well as the impacts of fishing bans on livelihood capital and the adaptation and recovery status after their withdrawal from fishing activities. Table 1 presents the geographical distribution of the sampled fishermen. In terms of provincial distribution, the percentages are as follows: Hunan Province accounts for 18.89%, Jiangsu Province for 27.21%, Jiangxi Province for 11.84%, Anhui Province for 13.85%, Sichuan Province for 13.6%, and Chongqing City for 14.61%. SPSS was utilized to conduct reliability and validity tests on the questionnaire data. The reliability analysis resulted in a Cronbach’s α coefficient of 0.830, suggesting a high level of data reliability. Regarding the validity test, the KMO value was 0.745, indicating its suitability for factor analysis. Bartlett’s test revealed an associated probability value below the significance level (p < 0.05), suggesting satisfactory validity of the model data. To preclude multicollinearity that might inflate the standard errors related to the estimated parameters, variance inflation factor tests were conducted. The VIF values were ≤ 2.312, indicating the absence of multicollinearity among variables, thereby enabling the effective implementation of regression analysis.

**Table 1:** Geographical distribution of the sample retired fishermen from China’s Yangtze River.

Provinces	City/district/county	Sample size	Percentage(%)	Provinces	City/district/county	Sample size	Percentage(%)
Hunan 75	Yuanjiang	11	0.15	Jiangsu 108	Sihong	21	0.19
	Hanshou	10	0.13		Xuyi	16	0.15
	Xiangyin	9	0.12		Jingjiang	13	0.12
	Anxiang	8	0.11		Yixing	11	0.10
	Heshan	7	0.09		Wujiang	10	0.09
	Taojiang	7	0.09		Jiangdu	9	0.08
	Junshan	6	0.08		Wujin	8	0.07
	Miluo	5	0.07		Hongze	7	0.07
	Nan county	5	0.07		Jiangyin	7	0.07
	Liuyang	4	0.05		Wuzhong	6	0.06
	Linxiang	3	0.04	Jiangxi 47	Poyang	17	0.36
Chongqing 58	Yunyang	14	0.24		Duchang	14	0.30
	Hechuan	12	0.21		Yugan	9	0.19
	Jiangjin	10	0.17		Xinjian	7	0.15
	Fuling	9	0.16	Anhui 55	Anqing	16	0.29
	Yubei	8	0.13		Wuhu	15	0.27
	Beibei	5	0.09		Huangshan	10	0.18
Sichuan 54	Fushun	37	0.69		Tongling	8	0.15
	Ziyang	17	0.31		Chizhou	6	0.11

### 3.2. Measures

A 5-point Likert-type scale was employed to assess each item. The initial section of the questionnaire gathered fundamental information from fishermen, encompassing their place of residence, gender, age, educational attainment, income level, primary income source, and means of livelihood. The second segment pertains to the measurement of variables associated with livelihood resilience. Risk perception was gauged by three items related to living standards, economic benefits, and development opportunities [8,42]. The standard of living comprises the extent to which individuals can avail themselves of government ecological policies, minimum income support programs, and major illness relief assistance. Economic benefits involve anticipations regarding changes in income and employment stability. Development opportunities refer to fishermen’s cognitions of access to opportunities, fluctuations in the external environment, and the alignment of personal capabilities with these opportunities [43]. The propensity to adopt ecological farming methods serves as the dependent variable in this research. This propensity is measured by the survey question: “During the process of livelihood transition or recovery, is your household likely to shift from other livelihood strategies to engage in ecological farming?” A positive response is assigned a value of “1”, while a negative response is assigned a value of “0”. The results of the descriptive analysis of the variables are presented in Table 2.

**Table 2:** Variable definition and descriptive statistics.

Latent variable	Variable code	Variable content	Variable assignment	Factor loading
Human capital	HC1 HC2 HC3	Number of family laborers (person) Educational level Health status of family members	0 = “1”,1-2 = “2”,3-4 = “3”,5-6 = “4”, ≥ 7 = “5” Below elementary school = “1”, Elementary school=”2”, Junior high school = ”3”, High school = ”4”, Above high school = ”5” Long-term illness = “1”, Often illness=”2”, Sometimes illness = ”3”, Rarely illness = ”4”, Healthy = ”5”	0.783 0.805 0.774
Physical capital	PC1 PC2 PC3	Number of household agricultural machinery The value of durable goods owned by household Per capita residential area(m2)	0 = “1”,1 = “2”,2 = “3”,3 = “4”,4 and more = “5” 10000 and below=“1”, 100000-30000=”2”, 30000–50000 = ”3”, 50000–80000 = ”4”, 80000 and above=”5” ≤10 m^2^ = “1”,10–20 m^2^ = “2”,20–30 m^2^ = “3”,30–40 m^2^ = “4”, ≥ 50 m^2^ = “5”	0.692 0.713 0.701
Natural capital	NC1 NC2	Contracted arable land area (mu) Area of owned aquaculture ponds (mu)	0 = “1”,0- 1 = “2”,1-3 = “3”, 3-5 = “4”, ≥ 5 = “5” 0 = “1”,0- 1 = “2”,1-3 = “3”, 3-5 = “4”, ≥ 5 = “5”	0.735 0.712
Financial capital	FC1 FC2 FC3	Annual household income after returning (10000 yuan) Proportion of non-agricultural income of fishermen before retirement Financial support (fishermen have insurance types)	5 = “1”,5-10 = “2”,10-20 = “3”,20-30 = “4”, ≥ 30 = “5” 20% = “1”,20% ~ 40% = “2”,40% ~ 60% = “3”,60% ~ 80% = “4”, ≥ 80% = “5” 0 = “1”,1 = “2”,2 = “3”,3 = “4”,4 and more = “5”	0.767 0.785 0.790
Social capital	SC1 SC2 SC3	Familiarity with neighbors after retirement Connection with government after retirement Frequency of participation in community	Basic unfamiliarity = “1”, Less familiarity=”2”, General = ”3”, Relatively familiar = ”4”, Very familiar = ”5” No contact = “1”, Less contact=”2”, General = ”3”, More frequent contact = ”4”, Frequent contact = ”5” 0 = “1”,1-2 = “2”,3-4 = “3”,5-6 = “4”, ≥ 7 = “5”	0.819 0.805 0.730
Self-organization ability	OA1 OA2 OA3	Level of understanding of the policy of changing production and employment Accessibility of government public resources Neighborhood effect of others’ choices	Very unfamiliar = “1”, Not familiar=”2”, Generally = ”3”, Familiar = ”4”, Very familiar = ”5” Inaccessible = “1”, Not very accessible=”2”, Generally = ”3”, Relatively accessible = ”4”, Accessible = ”5” Not affected = “1”, Not very affected=”2”, Generally = ”3”, Relatively affected = ”4”, Very affected = ”5”	0.826 0.880 0.775
Learning ability	LA1 LA2	The enthusiasm for participating in technical training Frequency of exchanging information	Never participate = “1”, Rarely participate=”2”, Sometimes participate = ”3”, Frequently participate = ”4”, Always participate = ”5” Never communication = “1”,Very little communication = “2”,Occasional communication = “3”,Frequent communication = “4”, Always communication = “5”	0.902 0.893
Regulating variable	R	Risk perception of retired fishermen	Obtained from the weighted average of risk perception of living standards, risk perception of economic benefits and risk perception of development chances	0.797
Dependent variable	Y	The willingness of retired fishermen to adopt ecological farming practices	The inclination to embrace ecological farming methodologies constitutes the dependent variable within the scope of this research. This inclination is quantified by the survey inquiry: “In the course of transitioning or recuperating from livelihood alterations, is your household inclined to transition from alternative subsistence strategies to engage in ecological farming?” An affirmative response is designated with the value “1,” whereas a negative response is assigned the value “0.”	0.817

### 3.3. Data analysis

Two methodologies were utilized to test our hypotheses: Partial Least Squares Structural Equation Modeling (PLS-SEM) via Smart PLS 4.0.8 and Fuzzy-set Qualitative Comparative Analysis (fs/QCA) through fs/QCA 3.0. The PLS-SEM technique is applied to examine the hypothesized relationships within causal frameworks and to evaluate their cumulative effect on one or more outcomes [8]. The validity of the PLS-SEM approach lies in its ability to assess the interrelationships among diverse latent variables while simultaneously minimizing errors in the model. Notably, the SmartPLS software proficiently employs PLS-SEM to scrutinize and validate structural equation models, enabling accurate determinations of path coefficients and significance levels between latent variables without imposing constraints on data distribution forms or quantities. This method is suitable for forecasting applications and theoretical construction and has gradually emerged as a prevalent technical tool in empirical research [44]. Therefore, the empirical analysis results of this study initially adopt PLS-SEM to verify the influencing factors related to the willingness of retired fishermen to adopt ecological farming practices. However, it can only capture the linear relationship between structures and fails to account for unanticipated reverse cases [45]. According to Ragin et al. (2008), the theoretical approach of fs/QCA is employed to conduct comparative case analyses and identify the “synergistic effects” of interactions among multiple conditional variables on specific outcomes [46]. This analytical methodology, based on set theory and Boolean logic, was specifically designed to examine social phenomena using small sample sizes [47,48]. The application of this method may contribute to the resolution of research issues related to livelihoods. Consequently, to overcome the limitations of PLS-SEM and enhance the understanding of the complex causal dynamics associated with the resilience of micro-subjects’ livelihoods and the intention to engage in ecological farming, the present study employs asymmetric fs/QCA as a supplementary research tool, integrating multiple equivalent configuration schemes.

Fs/QCA encompasses the subsequent steps. 1) Calibration: This procedure entails computing the mean value of each scale item and transforming the antecedents into fuzzy sets by utilizing fuzzy scores of 95%, 50%, and 5% [49]. 2) Analysis of necessary conditions: This is employed to validate whether each antecedent condition is indispensable for the generation of the outcome, which is a fundamental premise of fs/QCA. In the course of condition analysis, a condition is regarded as necessary when its consistency surpasses a significance level of 0.9 [50]. 3) Truth table analysis: This involves classifying all possible combinations and ranking the scenarios according to a minimum frequency of 1, a consistency threshold of 0.8, and a PRI consistency of 0.5 [51,52].

## 4. Results and analysis

### 4.1. Results of PLS-SEM

#### 4.1.1. Measurement model results.

The measurement model, encompassing reliability and validity, along with the structural model, is evaluated through the application of PLS-SEM [53]. Table 3 reveals that the Cronbach’s alpha and composite reliability (CR) values of all constructs surpass the recommended threshold of 0.7, and the average variance extracted (AVE) of each construct exceeds the suggested threshold of 0.5 [44,53]. Consequently, all the measurement models exhibit reliability and validity. Moreover, the hetero-trait-monotrait ratio (HTMT) was examined to determine discriminant validity [54]. All the HTMT values were below 0.9, thereby validating the discriminant validity [44,54].

**Table 3:** Reliability estimates,and convergent and discriminant validity.

Variables	Dimension reliability and validity			HTMT				
	Cronbach’s alpha	CR	AVE	RP	BA	OA	LA	EFI
Risk Perception(RP)	0.785	0.876	0.697					
Buffering Ability(BA)	0.732	0.833	0.593	0.512				
Self-organization Ability(OA)	0.790	0.852	0.615	0.558	0.673			
Learning Ability(LA)	0.809	0.865	0.627	0.607	0.695	0.765		
Ecological Farming Intention(EFI)	0.778	0.849	0.639	0.429	0.536	0.691	0.811	

#### 4.1.2. Structural model and hypothesis testing.

According to Hair et al., the coefficient of determination (R^2^),effect sizes (f^2^), and predictive relevance (Q^2^) all play a role in structural model assessment. The R^2^ values for buffering ability, self-organization ability, learning ability and ecological farming intention were 0.323, 0.416, 0.507 and 0.528, respectively, indicating that the variables of all the items explained variance. The results for f ^2^ showed that buffering ability (f^2 ^= 0.219) self-organization ability(f^2^ = 0.275) and learning ability(f^2^ = 0.320)had a medium effect size, while risk perception (f^2^ = 0.143) had a small effect size on ecological farming intention. The model’s Q^2^ values of buffering ability (Q^2^ = 0.197), self-organization ability (Q^2^ = 0.228), learning ability(Q^2^ = 0.256), and ecological farming intention (Q^2^ = 0.072) were all greater than zero, indicating they all had some predictive power.

As indicated in Table 4, in contrast to Hypothesis 1, risk perception exhibited a negative correlation with the ecological farming intentions of retired fishermen (β = ‒0.301, P < 0.05). Nevertheless, it displayed a positive correlation with buffering (β = 0.367, P < 0.05), self-organization (β = 0.474, P < 0.01), and learning abilities (β = 0.420, P < 0.001). Consequently, Hypotheses 6, 7, and 8 are corroborated. The path coefficient from livelihood resilience to the ecological farming intentions of retired fishermen was 0.445 (P < 0.001), signifying a positive direct relationship between these variables; thus, Hypothesis 5 is substantiated. Hypotheses 2, 3, and 4, which posited positive associations between buffering ability (β = 0.378, P < 0.01), self-organization ability (β = 0.459, P < 0.01), learning ability (β = 0.493, P < 0.001), and the outcome variable, were also substantiated. The mediating effects were examined through a bootstrapping procedure employing a resample size of 10,000 (refer to Table 5).The results indicate that in the impact path of risk perception on livelihood resilience, ecological farming intention of retired fishermen indirect effect size of 0.251 with a confidence interval of [0.069, 0.516] excluding 0, suggesting a significant indirect mediating effect; thus, Hd is supported. Moreover, buffering ability and livelihood resilience mediated the association between risk perception and ecological farming intention of retired fishermen (β = 0.209, T = 1.749, P < 0.05). Similarly, the mediating role of self-organization ability (β = 0.244, T = 2.656, P < 0.01) and learning ability (β = 0.275, T = 2.327, P < 0.001) between risk perception and ecological farming intention of retired fishermen is also significant, supporting for H_b_ and H_c_. However, the mediating role of buffering-self-organization-learning abilities between risk perception and livelihood resilience was not significant (β = 0.142, T = 1.861, P = 0.067); thus, H_e_ was not supported.

**Table 4:** Direct results of the structural model.

Hypothesis	f^2^	β	T value
1. Risk perception → Ecological farming intention of retired fishermen	0.143	−0.301*	2.121
2. Buffering ability → Ecological farming intention of retired fishermen	0.219	0.378**	2.051
3. Self-organization ability → Ecological farming intention of retired fishermen	0.275	0.459**	2.494
4. Learning ability → Ecological farming intention of retired fishermen	0.320	0.493***	2.506
5. Livelihood resilience → Ecological farming intention of retired fishermen	0.306	0.445***	2.372
6. Risk perception → Buffering ability	0.297	0.367*	3.073
7. Risk perception → Self-organization ability	0.319	0.474**	3.265
8. Risk perception → Learning ability	0.284	0.420***	3.371

Note: Effect sizes (ƒ2): small = 0.02, medium = 0.15, and large = 0.35; * means *P* < 0.05, ** means *P* < 0.01, *** means *P* < 0.001

**Table 5:** Assessment of mediating effects.

Hypothesis	β	T value	Confidence interval[25%,97.5%]
a. Risk perception→Buffering ability→Livelihood resilience→Ecological farming intention of retired fishermen	0.209*	1.749	[0.045,0.527]
b. Risk perception→Self-organization ability→Livelihood resilience→Ecological farming intention of retired fishermen	0.244**	2.656	[0.073,0.478]
c. Risk perception→Learning ability→Livelihood resilience→Ecological farming intention of retired fishermen	0.275***	2.327	[0.066,0.575]
d. Risk perception→Livelihood resilience→Ecological farming intention of retired fishermen	0.251***	2.087	[0.069,0.516]
e. Risk perception→Buffering ability→Self-organization ability→Learning ability→Livelihood resilience	0.142^nc^	1.861	[0.042,0.351]

Note: * means *P* < 0.05, ** means *P* < 0.01, *** means *P* < 0.001; nc = not significant, 25% = lower limit; 97.5% = upper limit

### 4.2. Results of fs/QCA

#### 4.2.1. Necessary condition analysis.

Firstly, data calibration is carried out to convert the range of measurement values into a scale spanning from 0 to 1, where 0 represents complete non-membership and 1 denotes complete membership [46,49]. When calibrating continuous variables, it is requisite to initially compute their mean values and subsequently employ the calibrate() function in fsQCA3.0 for data calibration based on the criteria of 5%, 50%, and 95% [51]. Considering that the livelihood resilience variable selected in this research is a five-category variable, anchor points are established via direct calibration methods in accordance with theoretical frameworks or data distributions [55]. In this study, three anchor points are ascertained using quantiles and professional expertise: specifically, the minimum value is calibrated as 0 (non-membership), the median value as 0.5 (cross-over point), and the maximum value as 1 (complete membership) [55].

More precisely, complete membership (1) clearly defines the conditions under which a case “fully belongs” to the target set. The cross-over point (0.5) establishes a fuzzy state of “partial membership”, which typically reflects a theoretical “threshold”. Complete non-membership (0) clearly delineates the characteristics of cases that “do not belong at all” [46,48]. In the research on “strong ecological farming willingness”, full affiliation can be defined as having a per-capita residential area of no less than 50 square meters, a total value of household durable goods exceeding 80,000 yuan, and engaging in highly frequent communication and interaction with the government. The driving intersection of willingness can be characterized by a per-capita residential area ranging from 20 to 30 square meters and a total value of household durable goods between 30,000 and 50,000 yuan, suggesting that these values are neither notably high nor low. Conversely, individuals with a per-capita residential area of no more than 10 square meters, who do not participate in activities or technical training and seldom communicate with other fishermen, are regarded as having no farming willingness.

For other types of variables, such as the education level variable, the categories are calibrated as follows: “below primary school” is calibrated to correspond to 0; “junior high school” is calibrated to correspond to 0.5; and “undergraduate and graduate” is calibrated to correspond to 1. In the case of health-related variables, “healthy” is calibrated as 1; “fair” is calibrated as 0.5; and “illness” is calibrated to correspond to 0. To prevent cases with a membership degree precisely equal to 0.5 from being excluded during the analysis, this study adjusts these calibrated values from 0.500 to 0.501 [56].

The fuzzy-set qualitative comparative analysis (fs/QCA) technique is employed to examine whether individual condition variables, including their logical negations [57], serve as necessary conditions for the willingness of retired fishermen to adopt ecological farming practices. The consistency level within fuzzy sets is selected as the measure of necessity, and its computational formula is presented as follows:


Consistency ((Xi≤Yi)=∑[min(Xi,Yi)]/∑(Xi) (1)

In equation (1), *X*_*i*_ and *Y*_*i*_ are the membership degrees of individual *i* in combinations *X* and *Y* respectively, and the consistency value ranges from 0 to 1. When the consistency exceeds 0.9, it is deemed that this condition is a necessary condition for the outcome [58]. Table 6 presents the results of calculating the necessary conditions for fishermens’ willingness to adopt ecological farming based on fsQCA 3.0. It is evident that the consistency across all conditions is below 0.9, suggesting that no essential factors are influencing fishers’ inclination to embrace ecological farming practices.

**Table 6:** Results of the necessity analysis of the antecedent conditions.

Condition variable	Retired fishermen have strong willingness to adopt ecological farming		Retired fishermen have weak willingness to adopt ecological farming	
	Consistency	Coverage	Consistency	Coverage
Livelihood capital	0.520	0.560	0.628	0.622
~ Livelihood capital	0.649	0.655	0.556	0.516
Subsidy benefits	0.603	0.621	0.601	0.569
~ Subsidy benefits	0.582	0.613	0.599	0.581
Policy awareness	0.693	0.605	0.732	0.587
~ Policy awareness	0.527	0.681	0.507	0.603
Neighbourhood trust	0.553	0.581	0.624	0.603
~Neighbourhood Trust	0.622	0.643	0.566	0.538
Technical Training	0.459	0.541	0.588	0.637
~ Technical Training	0.692	0.646	0.576	0.495
Information exchange	0.851	0.553	0.886	0.530
~Information Exchange	0.277	0.725	0.253	0.609
Risk Perception	0.753	0.674	0.640	0.581
~Risk Perception	0.526	0.623	0.582	0.697

Note:  ~ means negated (lack of the causal condition). For example, “~Livelihood capital” represents non-livelihood capital; that is, fishermens’willingness lack livelihood capital

#### 4.2.2. Conditional configuration adequacy analysis.

The results of the fuzzy-set qualitative comparative analysis (fs/QCA) yield three types of solutions, each characterized by a distinct level of complexity: complex solutions, parsimonious solutions, and intermediate solutions [59]. Generally, current research often reports on intermediate solutions, and simultaneously differentiates core conditions from peripheral conditions in conjunction with parsimonious solutions. Conditions that appear in both the parsimonious and intermediate solutions are defined as core conditions. In contrast, those that are unique to the intermediate solution are classified as peripheral conditions [60]. The configuration analysis results regarding fishermen’s willingness to adopt ecological farming are presented in Table 7.

**Table 7:** Configuration analysis of retired fishermen’s willingness to adopt ecological farming.

	Fishermen have strong willingness to adopt ecological farming			Fishermen have weak willingness to adopt ecological farming		
	Self-organization Dominant	Self-organization – Learning Driven	Buffering – Learning Driven	Buffering-Learning Inhibition	Self-organization – Learning Inhibition	Buffering – Self-organization Inhibition
Conditional Configuration	Configuration 1	Configuration 2	Configuration 3	Configuration 4	Configuration 5	Configuration 6
Livelihood Capital	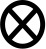	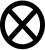	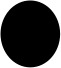	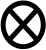		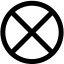
Subsidy benefits	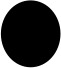	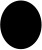	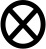		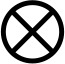	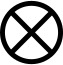
Policy awareness	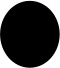		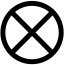	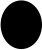	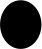	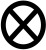
Neighborhood trust		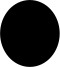	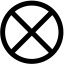		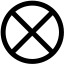	
Technical training		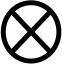	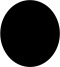	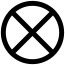	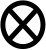	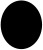
Information exchange	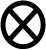	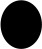	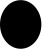	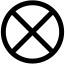	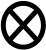	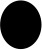
Risk perception	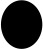	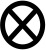		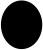		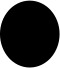
Consistency	0.771	0.815	0.796	0.827	0.758	0.819
Raw coverage	0.276	0.239	0.322	0.169	0.173	0.208
Unique coverage	0.081	0.125	0.159	0.113	0.099	0.162
Solution consistency	0.797			0.821		
Solution coverage	0.685			0.702		

Note: 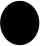
or 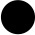 if the condition exists, 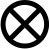 or 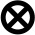 if the condition does not exist; 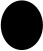 or 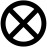
is the core condition, and 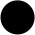
or 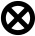 is the peripheral condition. Blank indicates that the condition can be present or absent.

(1)
**Configuration with strong willingness of fishermen to adopt ecological farming**

Table 7 presents three distinct pathways that impact fishermen’s propensity to adopt ecological farming practices. The consistency levels of both individual and collective solutions exceed 0.75, with the collective solution demonstrating a consistency level of 0.797. This implies that 79.7% of retired fishermen display a strong propensity to engage in ecological farming after ceasing fishing activities. The coverage of the collective solution is 0.685, signifying that these three conditional configurations account for 68.5% of cases marked by a strong propensity among fishermen to adopt ecological farming. These configurations can be regarded as sufficient combinations of conditions that foster a strong propensity among fishermen towards ecological farming. Upon further analysis, these conditional configurations disclose distinct pathways influenced by buffering, self – organization, learning, and risk perception, which affect fishermen’s propensity to adopt ecological farming.

**Fig 2:**
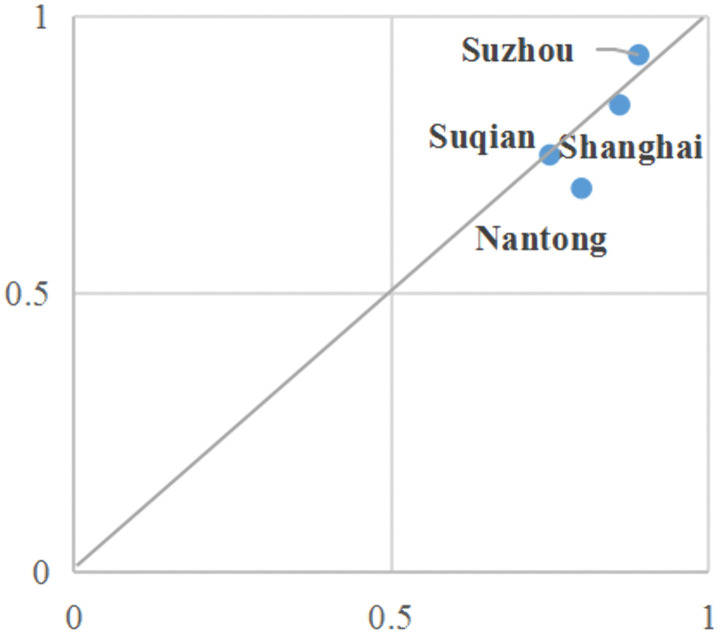
Case collection explained by configuration 1.

Self-organization Dominant. Configuration 1 expounds on the crucial role of subsidy benefits and policy awareness. In the context of fishermen’s propensity to adopt ecological farming, enhanced policy awareness and the perception of government subsidies can overcome the limitations imposed by buffering and learning conditions. The moderating effect of risk perception on the combination of self- organization ability can also promote fishermen’s inclination to participate in ecological farming. This is because self-organization ability provides social support for adaptation [21]. Fishermen with strong social integration skills have a higher sense of social support, enabling them to utilize social resources more effectively when facing challenges [25]. Consequently, effective risk perception strengthens the positive impact of self – organization ability on the adaptation inclination.The cases illustrated by Configuration 1 are mainly concentrated in counties along the lower reaches of the Yangtze River and relatively developed eastern counties. On one hand, these regions are equipped with more abundant economic resources and substantial financial subsidies, which alleviates their concerns when attempting to adopt ecological farming techniques. Moreover, the increased access to policy information and elevated cognitive capabilities help mitigate the negative effects of information asymmetry, commonly known as the “lemon market effect” [39], thereby effectively reducing perceived market risks. On the other hand, due to significant subsidies and strong market awareness, fishermen in these areas generally experience a more rapid restoration of their livelihoods. This enables them to promptly adjust their early production experiences and fishing habits, avoiding risks associated with unfamiliarity that may lead to yield reduction [42]. The consistency of this pathway configuration is 0.771, with a unique coverage of 0.081 and a raw coverage of 0.276. This indicates that this pathway can account for approximately 27.6% of cases where fishermen demonstrate a strong willingness. Additionally, approximately 8.1% of cases where fishermen are inclined to engage in ecological farming can be solely attributed to this pathway (Fig 2).

**Fig 3:**
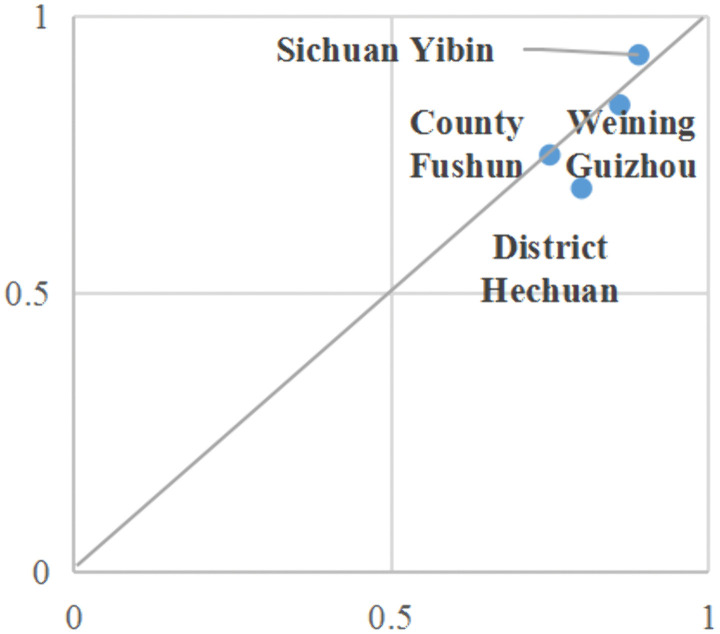
Case collection explained by configuration 2.

Self-organization-Learning Driven. In Configuration 2, neighborhood trust assumes a pivotal position, while subsidy benefits and information exchange function as supplementary elements. The cases illustrated by this conditional configuration primarily pertain to remote fishing villages located in the western regions of China and the upper reaches of the Yangtze River. These villages are often geographically isolated, with relatively weak foundations for aquaculture production and limited coverage of government support [61], which to some extent hinders the dissemination of ecological farming models. Neighborly interaction and shared communication have emerged as essential channels for fishermen to acquire information. Fishermen with strong social trust may be influenced by their peers, thereby enhancing their willingness to adopt ecological farming and generating a neighbor effect [27]. For example, fishermen from County F in Sichuan Province, China, reported that after ceasing fishing, they learned from local fishermen about job opportunities at an integrated rice-fish farming demonstration base, where they could earn wages and receive industry dividends at the end of the year [32]. In H District of Chongqing City, China, initiatives have been launched to promote simultaneous rice-shrimp cultivation to increase fishermen’s incomes. Leveraging the opportunities presented by high-standard farmland construction, this district has actively developed 23,000 acres of rice-shrimp farming areas involving 46 major operators [62]. The consistency score for this driving-path configuration is 0.815, with a raw coverage rate of 0.239; it accounts for approximately 23.9% of cases where fishermen demonstrate a strong willingness to adopt ecological farming (Fig 3).

**Fig 4:**
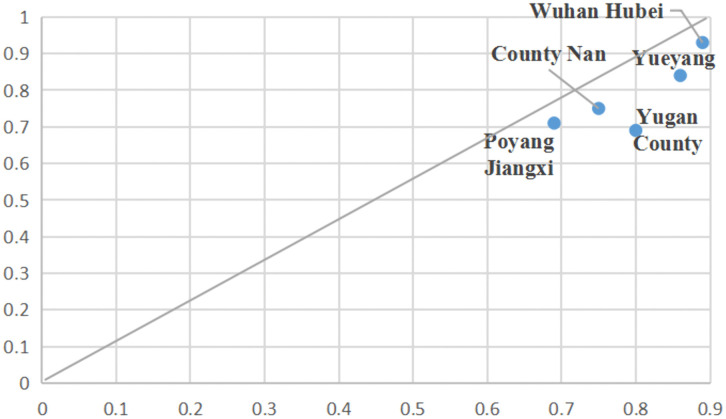
Case collection explained by configuration 3.

Buffering-Learning Driven. In Configuration 3, livelihood capital and technical training assume pivotal positions, while information exchange serves a complementary function. The consistency of this configuration stands at 0.796, with a raw coverage rate of 0.322 (Fig 4). Examples that can be explicated by Configuration 3 include the three provinces located in the middle reaches of the Yangtze River in China, namely Hunan, Jiangxi, and Anhui. Firstly, this region boasts significant natural advantages. The middle reaches of the Yangtze River serve as a major production base for staple grains in China, characterized by abundant water resources and favorable climatic conditions conducive to agricultural production, attributed to the concurrent occurrence of rainfall and heat [63]. The proportion of individuals previously engaged in farming who have transitioned to other forms of employment exceeds 60% [64].Secondly, government initiatives aimed at enhancing fishermen’s capital endowments safeguard their productive activities. This involves optimizing service systems to augment both material and financial capital for fishermen [5]. Additionally, endeavors are made to strengthen technical training for fishermen to elevate human capital levels through the dissemination of knowledge regarding production operations and refined management practices [24]. As a representative case study, County N in Hunan invited experts to deliver lectures on aquaculture knowledge and explored digital empowerment strategies utilizing new-media platforms such as big data to facilitate learning among local fishermen. In B Township of Anhui Province, training methods integrated indoor technical instruction with on-site guidance. Experts initially provided explanations on livestock and poultry farming, disease prevention, and the techniques for mugwort cultivation [26]. Meanwhile, the government of Y County in Jiangxi has incorporated retired fishermen into community management frameworks, coordinated education, health – care services, and housing security measures, which collectively enhance their livelihood capitals.

(2)
**Configuration of fishermen’s weak willingness to adopt ecological farming**

As shown in Table 7, three distinct pathways lead to the observed outcomes. Each pathway, together with the overall consistency of the solutions, exceeds 0.75, which implies that the antecedent variables have a strong explanatory power for the results. Moreover, the solution coverage rate is 0.702, suggesting that these configurational pathways account for 70.2% of the cases. Based on the core conditions within these pathways, the three identified types that influence fishermen’s reduced inclination towards ecological farming are: Buffering-Learning Inhibition (Configuration 4), Self- organization—Learning Inhibition (Configuration 5), and Buffering—Self-organization Inhibition (Configuration 6).

An analysis of these three categories indicates that buffering, self-organization and learning abilities are crucial factors influencing retired fishermen’s propensity to engage in ecological farming. The synergistic impact of buffering, self-organization, learning abilities, and risk perception in configurations 4 and 6 significantly affects fishermen’s inclination to participate in ecological farming.

Firstly, a substantial foundation of livelihood capital is indispensable for enhancing the likelihood of adopting ecological farming. The lack of sufficient human, physical, social, financial, and natural capital resources among fishermen significantly increases their vulnerability. Buffering ability provides substantial resource support for adjustments. Households with abundant resources have a stronger sense of livelihood security and are more optimistic about the potential benefits of ecological farming. Consequently, the prospect of higher returns enhances the positive influence of buffering ability on the inclination to adopt such practices.

Secondly, self-organization ability is of great significance in cultivating fishermen’s subjective intentions towards ecological farming. Meanwhile, within the context of interactions involving institutional policies and social networks, if fishermen encounter difficulties in enhancing their motivation for technology adoption through spontaneous or collective efforts, their self-organization capacities will be insufficient to strengthen their inclination towards ecological farming.

Finally, learning ability is crucial for cultivating farmers’ willingness to adopt eco-friendly farming methods. The acquisition of knowledge enables fishermen to master emerging technologies and understand the complexities of market dynamics. The improvement of their skill sets and understanding of market principles promotes a greater risk awareness within the fishing community, leading to higher expectations for the adoption of ecological farming practices. Therefore, fishermen with strong learning capacities are more capable of overcoming the barriers posed by risk.

Regardless of the degree of their inclination, fishermen’s risk perception significantly impacts the results of their estimations. This implies that individuals with a heightened risk awareness have a stronger ability to withstand risks. Under the influence of the risk temptation effect, they can leverage their inherent resources, self- organizational capabilities, and learning capacities to find solutions in dangerous situations, with the aim of achieving the desired returns. In essence, risk perception has a positive effect on enhancing livelihood resilience, especially regarding the inclination towards adopting ecological farming practices, thereby further validating Hypothesis 2.

(3)
**Exogeneity testing**

The present research employs the instrumental variable method to accurately evaluate the impact of livelihood resilience on fishermen’s propensity to engage in ecological farming. The study uses the average livelihood resilience of fellow fishermen within the same fishing community in the same year as an instrumental variable, referred to as the “Community Average Livelihood Resilience of Fellow Fishermen.” Generally, the livelihood resilience of other fishermen in a specific community can affect an individual fisherman’s resilience through channels such as policy networks and neighborhood culture. Nevertheless, this external resilience does not directly affect the individual’s propensity to adopt new practices, thus meeting the necessary conditions for an instrumental variable.

The study applies a two-stage IV-Probit model to tackle endogeneity problems, and the results are presented in Table 8. In the first stage, the F-statistic is 21.5785. Both the Wald tests and Anderson-Rubin tests reject the null hypothesis. These statistical results suggest that the “Community Average Livelihood Resilience of Fellow Fishermen” is correlated with individual fishermen’s livelihood resilience and is not a weak instrument.The second – stage regression results show that, after addressing endogeneity issues, livelihood resilience still has a positive impact on the adoption propensity at a significance level of 1%. Compared with previous regression results, our endogeneity tests do not change this basic conclusion.

**Table 8:** Results of endogeneity test on the impact of livelihood resilience on adoption willingness.

Variable	Instrumental Variable Probit	
	Stage One: Livelihood resilience	Stage Two: Willingness to adopt ecological farming
Community average livelihood resilience of fellow fishermen	0.6059***	
	(0.1513)	
Livelihood resilience		8.9450***
		(3.7361)
Control variable	controlled	controlled
Stage I F-value	21.5785	
AR testing	11.5723(P = 0.0022)	
Wald testing	7.3389(P = 0.0030)	
Observed values	397	

## 5. Discussion

### 5.1. Differences in fishermen livelihood resilience across different driving paths

Our aim was to explore the relationship between fishermen ecological farming strategies and livelihood resilience. The results of the study showed that fishermen who adopted different ability configurations of livelihood resilience had different ecological farming intentions, with their intentions represented as SL(Self-organization-Learning), BL(Buffering-Learning) and SD(Self-organization Dominant) three types. This suggests a positive interaction between fishermens’ adaptation strategies and their livelihood resilience along the Yangtze river basin. This finding is consistent with some case studies in other regions: Pagnani found that livelihood resilience can reduce the negative impacts of environmental change and increase the adaptation of fishermen in India [65]; and Mohammed et al. also showed that farmers with higher livelihood resilience tended to have higher transformation intention in Ghana [66]. Several studies focusing on small-scale fishers’ development have also found that for fishers, strengthening social connection or cognitive values are appropriate ways to influence small-scale fishers’ participation willingness [67], and self-organization ability may lead to better management strategies within fishing communities. The elastic subsidy mechanism, entailing the dynamic adjustment of fishing ban subsidy standards in accordance with hydrological conditions, and the establishment of a community co-management model via the formation of basin fishery committees, can offer a valuable reference for resolving the conflict between fishermen’s livelihoods and ecological protection in the Mississippi River Basin. However, it is important to note that livelihood resilience does not always interact positively with adaptation strategies, and different ability configurations that integrate risk variations may increase different exposure and sensitivity to livelihood strategies [68]. For example, Quandt found that low-wage nonfarm labor reduces resilience and subjective intention [69]. Therefore, more insight is needed into the linkages between fishermen adoption of ecological farming strategies, livelihood resilience, and risk perception impacts.

Fishermen livelihoods are vulnerable to environmental change because ecological farming activities are more sensitive to climate and resource impacts (droughts, floods, etc. often disrupt agriculture) [70],which may explain our finding that fishermen adopting ecological farming have the highest buffering ability. At the same time, fishermen who adopt the ecological farming tend to be more active in accessing information (BL fishermen strive to restore the livelihood capital and enhance the stock of livelihood capital at significantly higher rates compared to SL and SD fishermen), have better training application than other types of fishermen, and thus have the highest learning ability. Restoration Projects of the Four Major Rivers in South Korea and Ecological Fisheries of the Mekong River in Thailand adapt to environmental change by diversifying crops, changing farming patterns, etc. These behaviors can also reduce the vulnerability of agricultural production to climate [71], thus increasing the buffering ability of BL fishermen. In addition, policy promotion and social integration are important factors for SL fishermen to have higher self-organization and learning ability. The SL model can be used as a reference for the establishment of a multi-tiered transformation support system for European Union fishermen. This may entail the creation of a market-based compensation mechanism for the withdrawal of fishing vessels, the integration of training with regional ecological aquaculture planning, and the utilization of subsidy leverage to encourage environmentally-friendly farming practices.

### 5.2. Optimization of support policy combinations for fishermen in different regions

In Table 9, among the seven configurations analyzed, after consolidation, a total of three distinct patterns that stimulate the willingness for high-ecological farming transformation have been identified. 1. “Capital-Skills” Driven Model. In this configuration, both livelihood capital and skill acquisition serve as core factors in enhancing fishermen’s inclination towards adopting ecological farming practices.2. “Policy-Experience” Driven Model. The C5 configuration path places greater emphasis on social integration, suggesting that community support can effectively alleviate various external disturbances encountered after the transition. In contrast, C3 and C4 highlight that livelihood transformation and restoration are primarily accomplished through policy support. 3. “Capital-Policy-Community-Skills” Integration Model. Fishermen within this configuration necessitate an integrated approach encompassing livelihood capital, government assistance, community support, and skills training to attain higher resilience in their livelihoods and cultivate a willingness for ecological farming transformation.

**Table 9:** Heterogeneous configuration results of fishermen’s willingness to adopt ecological farming.

	Eastern Region (Jiangsu)		Central Region (Hunan Anhui Jiangxi)			Western Region (Sichuan Chongqing)	
Conditional configuration	Capital-Skill Driven (C1-C2)		Policy-Experience Driven Model (C3-C5)			Capital-Policy-Community-Skills Integration Model (C6-C7)	
Livelihood Capital	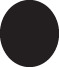	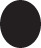	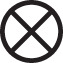				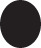
Subsidy Benefits		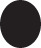		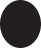	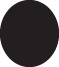	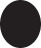	
Policy Awareness	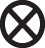		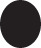	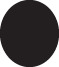			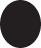
Neighborhood Trust		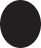	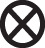		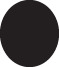	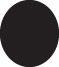	
Technical Training	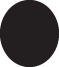	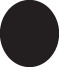	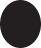	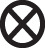	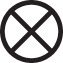	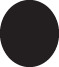	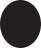
Information Exchange		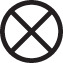	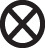	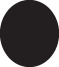	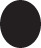		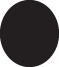
Consistency	0.901	0.854	0.839	0.935	0.908	0.872	0.867
Original Coverage	0.217	0.375	0.318	0.341	0.236	0.419	0.254
Unique Coverage	0.123	0.219	0.095	0.179	0.135	0.268	0.116

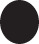
 or 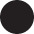 if the condition exists, 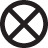 or 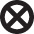 if the condition does not exist; 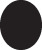 or 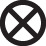 is the core condition, and or is the peripheral condition. 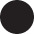 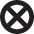 Blank indicates that the condition can be present or absent.

Owing to the heterogeneous impacts of geographical location, resource endowment, and other factors on fishermen’s willingness to make livelihood transitions, a configurational analysis is carried out for three distinct regions. Subsequently, a configurational effect analysis is performed to identify the policy path with the lowest support costs for the transition.

1) Analysis of support paths in eastern China and the lower Yangtze River region

In these regions, there are two effective pathways for fishermen to enhance their willingness. When both configurations can contribute to an increase in the local fishermen’s willingness for livelihood transition, it is viable to select the policy scheme with the lowest support costs. Configurations C1 and C2 attach great importance to skills training; nevertheless, C1 gives priority to compensating for livelihood capital, while C2 seeks assistance through minimum living guarantees and community support in addition to livelihood capital. Considering that most retired professional fishermen in the lower Yangtze River region have relatively singular income-generating capabilities and tend to continue engaging in primary industries such as agriculture or aquaculture after leaving fishing, there is a necessity to shift from simply providing “blood-transfusions” through policies to fostering self-sufficiency or “blood production”. Therefore, endeavoring to restore livelihood capital while guiding this group from traditional fishing practices towards ecological aquaculture is more in line with regional realities. Consequently, adopting a “capital-skills” driven approach is the optimal option.

2) Analysis of support paths in central China and the midstream Yangtze River region

Fishermen in central China have identified three effective approaches to elevate their inclination towards ecological farming transformation. When all three configurations can stimulate the transformation intentions of local fishermen, it is prudent to choose the policy alternative with the lowest support costs. These configurations must aid fishermen in obtaining adequate livelihood capital. Nevertheless, as local governments are required to bear part of the compensation funds for fishing cessation, restoring the already damaged livelihood capital within a short period entails substantial costs. Configurations C3-C5 emphasize “social integration” support measures that strengthen social belonging and participation among fishermen, thus establishing a robust foundation for sustainable livelihood development. Moreover, actively utilizing policy resources promotes the rapid recovery and improvement of livelihoods. Therefore, the “policy-experience” driven approach is of great significance for the livelihood transformation of fishermen in central regions.

3) Analysis of support paths in western China and the upstream Yangtze River region

For fishermen in this region, family endowments and endogenous development capabilities constitute the core elements of their livelihood transformation. Comprehensive support from diverse project funds is indispensable; otherwise, it fails to conform to the objectives of policy optimization. Therefore, for these fishermen to attain a successful transition, integration into the community is requisite to uphold regional social stability. Additionally, the government must establish safety nets for pensions and healthcare.Moreover, considering the relatively ample land resources available for development outside the protected areas in the upper reaches of the Yangtze River, part-time fishermen with certain land resources can notably augment their income through integrated farming practices. The difficulty of injecting livelihood capital is not significant. Nevertheless, if experience transfer and policy awareness are insufficient, they may emerge as critical constraints on the subsequent development of fishermen’s livelihoods.

## 6. Concluding remarks

### 6.1. Conclusions

This analysis led to two main conclusions: First, The PLS-SEM results show that livelihood resilience positively affect ecological farming willingness of retired fishermen. As indicated in the mediating path analysis, risk perception and livelihood resilience can positively influence ecological farming willingness of retired fishermen through buffering, self-organization and learning abilities. Second, the fs/QCA results demonstrated that simple antecedent variables do not constitute a necessary condition for promoting fishermens’ strong ecological farming willingness of retired fishermen, which depends on the conditions combined with another elements. There are three combination paths of strong willingness and three combination paths of weak willingness, in which livelihood resilience is essential for triggering strong ecological farming willingness. The inclination of fishermen to participate in ecological farming is affected by multiple factors, and the mechanisms for regional improvement exhibit substantial variations. Fishermen in three distinct regions are in urgent need of pursuing diverse pathways to realize livelihood transformation.

The distinctiveness of self-organization ability resides in its potential to circumvent the constraints of individual skills or resources via collective cooperation and social capital, consequently directly motivating and maintaining the inclination towards ecological farming. This “collective empowerment” mechanism suggests that fishermen can progressively refine their farming models through trial-and-error and innovation, without relying on systematic learning or external technological inputs. A low buffering ability typically implies inadequate resources for risk resistance; nonetheless, self-organization ability can diminish the reliance on external support by integrating internal resources or informal risk-sharing mechanisms, thereby independently facilitating ecological transformation. Therefore, based on the fs/QCA results, different combinations of factors in different scenarios have varying impacts on the ecological farming willingness of retired fishermen. This explains the complexity and situational nature of fishermens’ livelihood development.

### 6.2. Practical implications

First, in the context of ecological farming engagement, it is imperative to differentiate between fishermen who exhibit risk perception and those who do not. This differentiation originates from the disparate assessments conducted by these two groups regarding risk preferences, buffering ability, self-organizational skills, learning ability, and livelihood resilience. For fishermen with a lack of cognitive awareness, enhancing their propensity to participate is a prudent strategy. This can be achieved by emphasizing the economic benefits of ecological farming, the educational prospects it provides, and its positive impacts on living environments and living standards. For fishermen equipped with risk perception, it is equally essential to strengthen their commitment to sustainable practices in ecological farming. This approach will enable them to respond proficiently to disruptions, adapt to changes, and improve their diversified livelihood capabilities.

Second, we advocate for the implementation of differentiated policies guided by the synergy of “risk cognition—livelihood capital.” This entails devising a precise intervention mechanism that integrates risk-tiered subsidies with index-based insurance, thus converting fishermen’s internal awareness of market, technological, and environmental risks into external safeguards. For example, we propose the establishment of a specialized “Ecological Aquaculture Risk Buffer Fund”, which would set target prices and production volumes for products. Automatic compensation would be activated when market prices drop below these targets or when there are yield reductions due to disasters.Moreover, we aim to offer material subsidies for ecological resources to lower the initial costs for fishermen while promoting community-supported orders. This approach guarantees comprehensive protection across the entire supply chain, systematically enhancing resilience and incentivizing the adoption of ecological farming. By facilitating livelihood transformation through risk mitigation, this policy framework provides valuable perspectives for the sustainable development of resource-dependent communities within watershed management initiatives.

Finally, provincial governments along the Yangtze River ought to devise supportive policies for ecological farming in accordance with their specific situations, implementing a “localized” strategy. For fishermen in the eastern region, the implementation of “livelihood capital-based” compensation measures should be prioritized. It is suggested that the government augment the stock of livelihood capital for fishermen by increasing compensation for natural and material capital. For instance, fisheries transition funds can be provided, and subsidies for the upgrading of fishing facilities can be offered. For fishermen in central China, it is essential to enhance the support provided by policies and implement a “policy-driven” assistance strategy.This ensures that they comprehensively comprehend the policy content and actively leverage available resources to attain rapid recovery and enhancement of their livelihoods. Moreover, communities can regularly organize volunteer service activities for fishermen or hold diverse cultural events to facilitate communication and interaction among fishermen and between fishermen and the external world. For fishermen in the western region, the government ought to not only reinforce infrastructure construction and offer comprehensive information services but also augment their sense of social belonging and participation via “social integration” support measures. The local government at the grassroots level should guide the cultivation of a harmonious rural cultural ambiance and family relationship dynamics. This will empower fishermen to obtain resource support through this medium, thus establishing a robust network of resource relationships among fishermen, their families, and society as a whole to effectively address future changes. Simultaneously, it is essential to further enhance the pertinence and effectiveness of skills training by accurately identifying fishermen’s career aspirations and skill gaps. Meanwhile, a feedback mechanism should be established to monitor training results, facilitating the timely optimization of training programs based on this feedback.

## Supporting information

S1 Data **Follow-up survey data on fishermen affected by the fishing ban in ‘ten provinces, hundreds of counties, thousands of households’ along the Yangtze River.** This study centers around the overarching objective of ensuring that fishermen affected by the ban can ‘successfully transition out, maintain stable livelihoods, and attain prosperity.’ By employing a scientific approach to sample selection, we aim to dynamically capture essential information regarding the production and living conditions of those who have moved ashore. This data will provide robust support for a scientific evaluation of both the implementation status and effectiveness of various policies concerning fishing bans and withdrawals.
(XLSX)
